# Preconditioning of Rat Bone Marrow-Derived Mesenchymal Stromal Cells with Toll-Like Receptor Agonists

**DOI:** 10.1155/2019/7692973

**Published:** 2019-08-19

**Authors:** Fabiana Evaristo-Mendonça, Gabriela Sardella-Silva, Tais Hanae Kasai-Brunswick, Raquel Maria Pereira Campos, Pablo Domizi, Marcelo Felippe Santiago, Ricardo Augusto de Melo Reis, Rosalia Mendez-Otero, Victor Túlio Ribeiro-Resende, Pedro Moreno Pimentel-Coelho

**Affiliations:** ^1^Instituto de Biofísica Carlos Chagas Filho, Universidade Federal do Rio de Janeiro, Rio de Janeiro, RJ 21941-902, Brazil; ^2^Centro Nacional de Biologia Estrutural e Bioimagem (CENABIO), Universidade Federal do Rio de Janeiro, Rio de Janeiro, RJ 21941-902, Brazil; ^3^Instituto Nacional de Ciência e Tecnologia em Medicina Regenerativa, Rio de Janeiro, RJ 21941-902, Brazil; ^4^Núcleo Multidisciplinar de Pesquisa em Biologia (Numpex-Bio), Campus de Duque de Caxias Geraldo Guerra Cidade, Universidade Federal do Rio de Janeiro, Duque de Caxias, RJ 25255-030, Brazil

## Abstract

Bone marrow-derived mesenchymal stromal cells (BM-MSCs) are dynamic cells that can sense the environment, adapting their regulatory functions to different conditions. Accordingly, the therapeutic potential of BM-MSCs can be modulated by preconditioning strategies aimed at modifying their paracrine action. Although rat BM-MSCs (rBM-MSCs) have been widely tested in preclinical research, most preconditioning studies have employed human and mouse BM-MSCs. Herein, we investigated whether rBM-MSCs modify their phenotype and paracrine functions in response to Toll-like receptor (TLR) agonists. The data showed that rBM-MSCs expressed TLR3, TLR4, and MDA5 mRNA and were able to internalize polyinosinic-polycytidylic acid (Poly(I:C)), a TLR3/MDA5 agonist. rBM-MSCs were then stimulated with Poly(I:C) or with lipopolysaccharide (LPS, a TLR4 agonist) for 1 h and were grown under normal culture conditions. LPS or Poly(I:C) stimulation did not affect the viability or the morphology of rBM-MSCs and did not modify the expression pattern of key cell surface markers. Poly(I:C) did not induce statistically significant changes in the release of several inflammatory mediators and VEGF by rBM-MSCs, although it tended to increase IL-6 and MCP-1 secretion, whereas LPS increased the release of IL-6, MCP-1, and VEGF, three factors that were constitutively secreted by unstimulated cells. The neurotrophic activity of the conditioned medium from unstimulated and LPS-preconditioned rBM-MSCs was investigated using dorsal root ganglion explants, showing that soluble factors produced by unstimulated and LPS-preconditioned rBM-MSCs can stimulate neurite outgrowth similarly, in a VEGF-dependent manner. LPS-preconditioned cells, however, were slightly more efficient in increasing the number of regrowing axons in a model of sciatic nerve transection in rats. In conclusion, LPS preconditioning boosted the production of constitutively secreted factors by rBM-MSCs, without changing their mesenchymal identity, an effect that requires further investigation in exploratory preclinical studies.

## 1. Introduction

Mesenchymal stromal cells (MSCs), multipotent cells that can be isolated from a wide range of fetal, perinatal, and adult tissues, have emerged as a promising cell type for regenerative medicine [1, 2]. Although MSCs do not differentiate into neurons and glial cells in vivo, they have potent immunomodulatory properties and contribute to neural regeneration in a paracrine manner, which makes them an attractive option for the development of novel therapies for several neurological disorders [3–7].

One of the most interesting characteristics of MSCs is their ability to respond to changes in their environment, such as shifts in oxygen tension or exposure to pathogen-associated molecular patterns (PAMPs), alarmins, and other inflammatory mediators [8–11]. For instance, human, equine, and murine MSCs obtained from different tissues have been shown to express functional Toll-like receptors (TLR) [12–16]. Stimulation of MSCs with lipopolysaccharide (LPS), a typical TLR4 agonist, activates nuclear factor-kappa B and mitogen-activated protein kinase signaling pathways, resulting in the modulation of MSC paracrine activities [15, 17–20]. In addition, MSCs acquire a distinctive functional phenotype when stimulated with polyinosinic-polycytidylic acid (Poly(I:C)), a synthetic analog of double-stranded RNA that activates TLR3 [13, 19, 20]. These observations have led to the use of TLR agonists in preconditioning protocols aimed at boosting and/or modifying the therapeutic effects of MSCs [21–23].

To our knowledge, however, only a few studies have examined the effects of LPS and Poly(I:C) on rat bone marrow-derived MSCs (rBM-MSCs) [24–27], although rBM-MSCs are still widely used in preclinical studies. In a recent review article, for example, we identified 18 articles that have transplanted bone marrow-derived MSCs (BM-MSCs) in animal models of intracerebral hemorrhage and subarachnoid hemorrhage, 14 of which used rBM-MSCs [3]. Similarly, rBM-MSCs have been widely used in studies that have investigated the preclinical efficacy of BM-MSCs for the treatment of central and peripheral nerve disorders [4, 28]. Moreover, although most of these studies have provided evidence that BM-MSC-based therapies can promote the repair of peripheral nerve injuries [29–31], data on the effects of preconditioned MSCs on the peripheral nervous system are limited [21, 32]. In this study, we assessed the effects of a brief exposure to LPS or Poly(I:C) on the phenotype, paracrine/trophic activity, and proregenerative capacity of rBM-MSCs.

## 2. Materials and Methods

### 2.1. Animals

All procedures were approved and conducted in accordance with the Animal Care and Use Committee at the Universidade Federal do Rio de Janeiro. All animals received humane care in compliance with the “Principles of Laboratory Animal Care” formulated by the National Society for Medical Research and the U.S. National Academy of Sciences Guide for the Care and Use of Laboratory Animals. Wistar rats of both sexes weighing 250–400 g were used in this study.

### 2.2. rBM-MSC Culture and Maintenance

Wistar rats were deeply anesthetized via an intraperitoneal injection of a mixture of xylazine hydrochloride (15 mg/kg) and ketamine hydrochloride (100 mg/kg) and euthanized by cervical dislocation. Tibias and femurs of both hind paws were removed and carefully dissected from adjacent tissues in a sterile environment. The epiphyses were cut, and the bone marrow was flushed from the bones after centrifugation at 300 g for 5 min. Cells were resuspended in Dulbecco's modified Eagle's medium/Nutrient Mixture F-12 (DMEM/F12; Thermo Fisher Scientific) culture medium supplemented with 10% fetal bovine serum (FBS; Gibco) and were mechanically dissociated. Cells from one tibia and one femur were pooled and plated together in DMEM/F12 supplemented with 10% FBS (maintenance medium) in 100 mm cell culture dishes, which were kept in an incubator with 5% CO_2_ at 37°C. After 12 h, nonadherent cells were removed by washing twice with phosphate-buffered saline (PBS), and the maintenance medium was added and renewed every 2–3 days. When rBM-MSCs reached approximately 80–90% confluence, cells were harvested with a Trypsin/EDTA solution (0.25% Trypsin with 1 mM EDTA; Thermo Fisher Scientific) and increased by 3–5 passages in maintenance medium. The identity of rBM-MSCs was confirmed by evaluating the expression of cell surface markers by flow cytometry, as shown below, as well as by assessing their capacity to differentiate into cells of the mesoderm lineage, as demonstrated in our previous study [33].

### 2.3. RNA Isolation, cDNA Preparation, and RT-PCR

rBM-MSCs were cultured under standard conditions in 100 mm cell culture dishes for 24 h, harvested, and stored at –80°C until RNA preparation. RNA was isolated using the RNeasy Mini kit (QIAGEN) according to the manufacturer's instructions. 1 *μ*g total RNA was digested using DNase I (Ambion, Thermo Fisher Scientific), and cDNA was synthesized using SuperScript II Reverse Transcriptase (Thermo Fisher Scientific). RT-PCR was performed using the primers listed in Table 1 (20 pmol) and 40 cycles (95°C for 15 s, 56°C for 30 s, and 72°C for 30 s). The electrophoresis was carried out using PCR products in 2% agarose gel stained with GelRed Nucleic Acid Gel Stain (Biotium) and visualized with the Odyssey® Fc Imaging System, using Image Studio 5.x software (LI-COR Biosciences).

### 2.4. Poly(I:C) Internalization

To determine whether rBM-MSCs are capable of internalizing Poly(I:C), cells were plated at a density of a 10^3^ cells/35 mm glass-bottom dish (well size: 14 mm). After 24 h, cells were incubated with rhodamine-labeled high-molecular-weight Poly(I:C) (1 *μ*g/mL; InvivoGen) for the observation of the process of Poly(I:C) internalization using a Cell Observer spinning-disk confocal microscope (Zeiss).

### 2.5. Preconditioning of rBM-MSCs

The preconditioning protocol used in this study (TLR agonist concentration and incubation time) was based on the protocol described by Waterman et al. [20]. After 24 h in culture, rBM-MSCs were stimulated for 1 h with either LPS (10 ng/mL; LPS-B5 Ultrapure from *Escherichia coli* 055:B5; InvivoGen) or high-molecular-weight Poly(I:C) (1 *μ*g/mL; InvivoGen) diluted in maintenance medium. In the control group, rBM-MSCs were incubated with fresh maintenance medium for 1 h. Then, cells were carefully washed 5x with DMEM/F12 and cultured in maintenance medium without the stimulus for an additional 23 or 47 h, as specified below, for the evaluation of their phenotype, viability, and paracrine capacity.

### 2.6. Flow Cytometry Analysis

The immunophenotype of rBM-MSCs was evaluated by flow cytometry. MSCs were cultured under standard conditions in 100 mm cell culture dishes for 24 h and were then stimulated with LPS, Poly(I:C), or fresh maintenance medium for 1 h, washed 5x with DMEM/F12, and allowed to grow for 23 h with DMEM/F12+10% FBS, as described above. Briefly, rBM-MSCs were harvested and incubated with mouse anti-rat CD32 antibodies for 20 min (Cat# 550271; RRID: AB_393568, BD Pharmingen) for FcR blocking. Afterward, rBM-MSCs were incubated with the following antibodies, diluted (1 : 50) in PBS supplemented with 0.5% bovine serum albumin (BSA), for 20 min at 4°C: mouse anti-CD34 PE conjugated (Cat# sc-7324; RRID: AB_2009969; Santa Cruz Biotechnology), mouse anti-CD90 PE-Cy5 conjugated (Cat# 555597; RRID: AB_395971; BD Pharmingen), hamster anti-CD29 FITC conjugated (Cat# 555005, RRID: AB_395639; BD Pharmingen), or mouse anti-CD45 FITC conjugated (Cat# MR6901; RRID: AB_1476185; Caltag Laboratories). Cells were then washed with PBS+0.5% BSA and centrifuged at 300 g for 5 min. The pellet cells were again suspended in 300 *μ*L PBS, and data were acquired. Cell viability was evaluated by 7-AAD (Cat# 559925; BD Pharmingen). Data were acquired on a BD FACSAria II (BD Pharmingen), and the analyses were performed using FlowJo software version 10 (FlowJo, LLC).

### 2.7. Morphological Analysis of rBM-MSCs

rBM-MSCs were plated on 24-well plates (TPP) with glass coverslip inserts (Knittel Glaser) at a density of 5 × 10^4^ cells per well. After 12 h, cells were subjected to the preconditioning protocol for 1 h, washed 5x with DMEM/F12, and grown for additional 23 h in maintenance medium. Then, cells were washed 3x with PBS and fixed with 4% paraformaldehyde (PFA) for 10 min. For phalloidin staining, the following steps were performed: permeabilization with 0.1% Triton X-100 (Sigma-Aldrich) in PBS for 5 min, blocking with 0.5% BSA in PBS for 20 min, incubation with phalloidin conjugated with Alexa Fluor® 555 (diluted at 1 : 100 in PBS; Invitrogen) for 20 min, and a final washing step with PBS. Cells were mounted on slides with mounting medium containing 4′,6-diamidino-2-phenylindole (DAPI; Fluoroshield™, Sigma). All steps were performed at room temperature. Images were captured using an EVOS™ FL Cell Imaging System (Thermo Fisher Scientific).

### 2.8. Cell Viability Assay

The LIVE/DEAD cell viability assay (Cat# L3224; Thermo Fisher Scientific) was used to assess the viability of rBM-MSCs subjected to the preconditioning protocol. rBM-MSCs were plated on 24-well plates at a density of 5 × 10^4^ cells per well and cultured for 24 h. Then, cells were submitted to the preconditioning protocol for 1 h, washed 5x with DMEM/F12, and cultured in maintenance medium. After 23 h, the supernatant was discarded; cells were washed 3x with PBS and incubated in a PBS solution containing calcein-AM (1 : 5000) and ethidium homodimer-1 (1 : 1000) for 10 min at 37°C. Epifluorescence images from three randomly chosen fields per well were immediately taken using an EVOS FL Cell Imaging System. The ratio between the viable and total cells was calculated for each field.

### 2.9. Luminex® Assays

Cells were plated on 24-well plates and allowed to adhere for 24 h. Then, rBM-MSCs were stimulated with LPS or Poly(I:C) for 1 h as described above, washed 5x with DMEM/F12 medium, and grown in maintenance medium. After 23 or 47 h, the conditioned medium was collected and stored at –80°C. Levels of interferon- (IFN-) *γ*, interleukin- (IL-) 1*β*, IL-2, IL-4, IL-6, IL-10, IL-13, IL-18, tumor necrosis factor- (TNF-) *α*, and vascular endothelial growth factor (VEGF) were measured in undiluted samples, using the Magnetic Luminex Screening Assay (LXSARM-10; R&D Systems), following the manufacturer's instructions. Levels of VEGF and monocyte chemoattractant protein-1 (MCP-1) were analyzed with the Milliplex MAP Rat Cytokine/Chemokine Magnetic Bead Panel (RECYTMAG-65K; Millipore), in diluted (1 : 5) samples, following the manufacturer's instructions. Assay plates were immediately read and analyzed in a Luminex 200™ system (Millipore). All samples and standards were measured in duplicate.

### 2.10. Dorsal Root Ganglia (DRG) Explant Culture

DRG explants were obtained from postnatal day 1 C57BL/6J mouse pups. Cold-anesthetized animals were quickly decapitated. DRG were dissected and then incubated in DMEM/F12 culture medium containing nerve growth factor (NGF; 20 ng/mL, Thermo Fisher Scientific) for 24 h at 37°C and 5% CO_2_ in a 24-well plate (TPP) with coverslips (Knittel Glaser) previously coated with poly-D-lysine (10 *μ*g/mL, Sigma) and laminin (50 *μ*g/mL, Thermo-Fisher Scientific) on the bottom of each well. Then, DRG explants were incubated for 24 h with the conditioned medium from unstimulated or LPS-preconditioned rBM-MSCs (collected 47 h after stimulation with LPS for 1 h, as described above; diluted at 1 : 1 in fresh DMEM/F12 medium) or with fresh control medium (DMEM/F12+5% FBS, corresponding to the FBS concentration in the other conditions). Fresh control medium supplemented with 5% FBS and NGF (20 ng/mL) was used as a positive control. We used rBM-MSCs obtained from only 1 animal to reduce donor-related variability. Axitinib (5 *μ*g/mL, Sigma-Aldrich), a VEGFR inhibitor [34], was used to determine the potential effect of MSCs-derived VEGF on DRG neurite outgrowth. After incubation, the culture system was gently washed with PBS and fixed with 4% PFA, followed by immunostaining for Tuj1 (rabbit polyclonal anti-beta Tubulin 3/TuJ1 antibody; 1 : 300; Cat# GTX50789; RRID: AB_11171559; GeneTex). Alexa Fluor 488 donkey anti-rabbit IgG (H+L) (1 : 600; Cat# A-21206; RRID: AB_2535792; Thermo Fisher Scientific) was used as the secondary antibody. Samples were mounted directly on a microscope slide with Vectashield® mounting medium with DAPI (Vector Laboratories) and imaged on a Zeiss Axio Imager M2 microscope equipped with an Apotome System and fluorescence optics (Zeiss). Neurite density was quantified in a blinded fashion with ImageJ 1.48v software (National Institutes of Health, Bethesda, MD).

### 2.11. Sciatic Nerve Transection and rBM-MSC Transplantation

Total transection and reconnection of the sciatic nerve were performed as described previously [33, 35]. Male Wistar rats were deeply anesthetized via intraperitoneal injection of a mixture of xylazine hydrochloride (15 mg/kg) and ketamine hydrochloride (100 mg/kg). A small incision was made in the skin, and the right sciatic nerve was exposed at a midthigh level. The sciatic nerve was transected, and the distal and proximal stumps were reconnected to both ends of a silicone tube, leaving a gap of 4 mm between the stumps. 5 × 10^5^ rBM-MSCs (or preconditioned rBM-MSCs), suspended in 15 *μ*L of a Matrigel® Growth Factor Reduced Basement Membrane Matrix (Corning) solution (1 : 3 in DMEM/F12 medium), were injected into the tube. For these experiments, rBM-MSCs were grown for 24 h in DMEM/F12+10% FBS in tissue-culture dishes (100 mm), stimulated with LPS, Poly(I:C), or DMEM/F12 medium for 1 h, washed 5x with DMEM/F12, and harvested using a 0.25% Trypsin/EDTA solution (Thermo Fisher Scientific). We used rBM-MSCs obtained from only 1 animal to reduce donor-related variability. In the control group, only the Matrigel® solution was injected into the tube. After recovering from anesthesia, animals were returned to the animal facility and housed with free access to water and food.

### 2.12. Immunofluorescence Analysis of the Sciatic Nerve

Four weeks after surgery, rats were deeply anesthetized as described above and then transcardially perfused with ice-cold 0.9% saline for 5 min, followed by 4% PFA in phosphate buffer, pH 7.4, for 25 min, at a constant flow rate of 2 mL/min. The sciatic nerves were kept in 4% PFA for 12 h at 4°C and were cryoprotected via sequential immersion in 10%, 20%, and 30% phosphate-buffered sucrose solutions for 24 h at 4°C in each solution. The sciatic nerves were embedded in Tissue-Tek® O.C.T. compound (Sakura), and 14 *μ*m thick longitudinal sections were obtained using a cryostat (Leica CM 1850, Leica Microsystems). The sections were mounted on gelatin-coated slides and stored at −20°C until immunofluorescence procedures were conducted.

For immunofluorescence analysis, nerve sections were fixed with 4% PFA for 15 min, washed with PBS (3 × 5 min each), incubated with 0.3% Triton X-100 (Sigma-Aldrich) in PBS solution for 5 min, and blocked with 1% BSA in PBS solution for 30 min at room temperature. Then, nerve sections were incubated with rabbit anti-beta Tubulin 3/TuJ1 polyclonal antibody (1 : 300; Cat# GTX50789, RRID: AB_11171559; GeneTex) at 4°C overnight. Sections were then washed with PBS (3 × 5 min) and incubated with biotin-conjugated mouse anti-rabbit IgG (gamma-chain specific) monoclonal antibody (1 : 600; Cat# B5283; RRID: AB_258574; Sigma-Aldrich) for 90 min at room temperature. Then, sections were incubated with Cy3-conjugated streptavidin (1 : 100; Thermo Fisher Scientific) for 90 min at room temperature, washed with PBS (3 × 5 min), and sealed with mounting medium containing DAPI (Fluoroshield™; Sigma-Aldrich).

The regenerated portion of the sciatic nerve that was inside the polyethylene tube, stained for TuJ1, an axonal marker, was photographed using a 40x objective on a Zeiss Axio Imager M2 microscope equipped with an Apotome System and fluorescence optics (Zeiss). Images were analyzed in a blinded fashion using Zen software (Zeiss), by tracing 10 lines, 25 *μ*m from each other, on the proximal and distal portions of the regenerated nerve segment (500 × 250 *μ*m each), starting 20 *μ*m from the sutured region of the proximal stump. The number of TuJ1-positive axons crossing each line was averaged for each portion.

### 2.13. Statistical Analysis

Statistical analysis was performed using GraphPad Prism version 6.01 (GraphPad Software). Data are expressed as the mean ± SD or as the median with an interquartile range, as indicated in the figure legends. The following tests were used: one-way analysis of variance (ANOVA) with the Newman-Keuls multiple comparison test (for parametric data), Kruskal-Wallis test with Dunn's multiple comparison test (for non-parametric data), or the Friedman test with Dunn's multiple comparison test (for pairwise comparisons), as indicated. The observed differences were considered significant when *p* < 0.05.

## 3. Results

### 3.1. rBM-MSCs Express Innate Immune Receptors and Can Internalize Poly(I:C)

**Figure 1:**
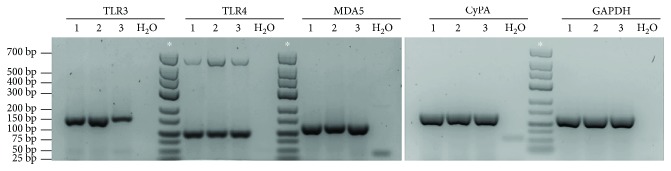
TLR3, TLR4, and MDA5 mRNA expression in rBM-MSCs. Agarose gel electrophoresis of reverse transcription-polymerase chain reaction (RT-PCR) products for the detection of TLR3, TLR4, and MDA5 mRNA in rat bone marrow-derived mesenchymal stromal cells (rBM-MSCs) from three different animals (1, 2, and 3). DNA molecular size markers were used (∗). Negative control: RT-PCR in the absence of RNA (H_2_O). Internal controls: cyclophilin A (CyPA) and glyceraldehyde 3-phosphate dehydrogenase (GAPDH). bp: base pairs.

Before proceeding with the preconditioning protocol, we evaluated whether rBM-MSCs express innate immune receptors for LPS (TLR4) and Poly(I:C) (TLR3 and MDA5) [36, 37] by RT-PCR. This analysis showed that rBM-MSCs from three different donors expressed the mRNA of TLR4, TLR3, and MDA5 (Figure 1).

**Figure 2:**
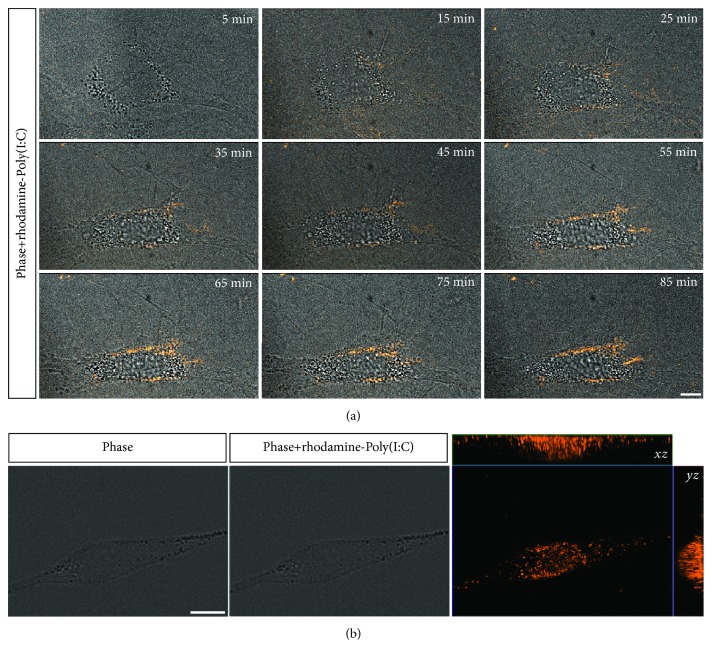
Internalization of rhodamine-conjugated polyinosinic-polycytidylic acid (Poly(I:C)) by rBM-MSCs. (a) Representative photomicrographs from time-lapse video microscopy, each corresponding to the indicated time points following the addition of rhodamine-conjugated Poly(I:C) to the culture medium, showing the overlay of phase contrast and spinning disk confocal fluorescence images of one cell (rhodamine-conjugated Poly(I:C) is shown in red). Scale bar: 10 *μ*m. (b) On the left, phase contrast image of a cell at 1 h after incubation with rhodamine-conjugated Poly(I:C). On the center, overlay of the phase contrast image and the maximum intensity projection of a z stack showing rhodamine-conjugated Poly(I:C) in red. On the right, orthogonal projections (XZ, YZ) of the z stack showing the internalization of rhodamine-conjugated Poly(I:C) (in red) by this cell. Scale bar: 10 *μ*m.

Given that TLR3 and MDA5 are intracellular receptors that recognize double-stranded RNA, Poly(I:C) need to be internalized to activate these receptors. We therefore performed an experiment to assess whether Poly(I:C) would be internalized by rBM-MSCs during the 1 h preconditioning protocol. Rhodamine-conjugated Poly(I:C) was added to the culture medium, and the cells were imaged with a confocal spinning disc microscope. Fluorescent spots were visible in MSCs within 15 min after incubation, and the number of fluorescent spots increased over the 85 min observation period (Figure 2A). Confocal reconstruction demonstrated the presence of intracellular Poly(I:C) in rBM-MSCs at 1 h (Figure 2(b)).

### 3.2. LPS and Poly(I:C) Preconditioning Do Not Affect the Phenotype and Viability of rBM-MSCs

**Figure 3:**
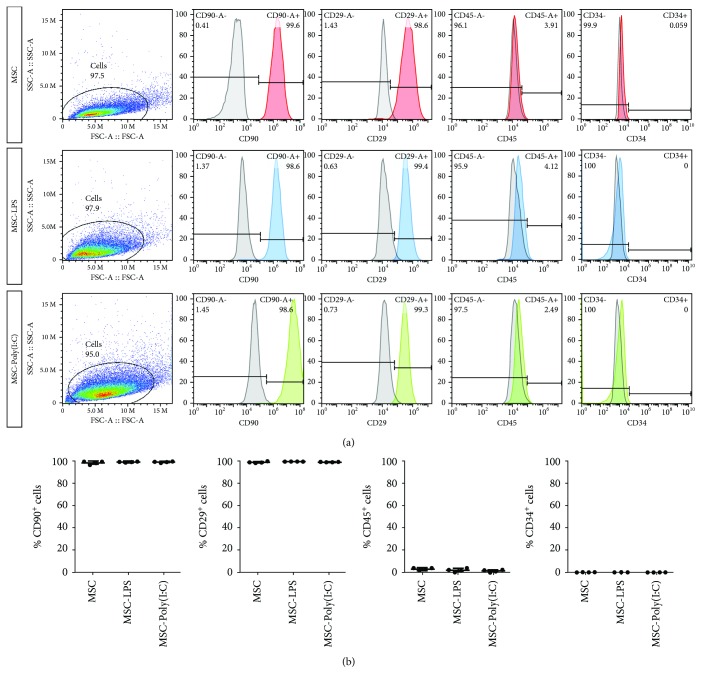
Flow cytometry analysis of the expression of cell surface markers in rBM-MSCs. Preconditioning with polyinosinic-polycytidylic acid (Poly(I:C)) or lipopolysaccharide (LPS) did not change the phenotype of rat bone marrow-derived mesenchymal stromal cells (rBM-MSCs). (a) Representative immunophenotype data for rBM-MSCs 23 h after a brief stimulation (1 h) with LPS (MSC-LPS) or Poly(I:C) (MSC-Poly(I:C), compared to unstimulated cells (MSC). Histograms show cells stained with isotype-matched control antibodies in gray and cells stained with antibodies of interest in color. On the left, dot plot representations showing the distribution of cell populations. FSC: forward scatter; SSC: side scatter. (b) Quantitative analysis showing that rBM-MSCs expressed CD90 and CD29 (≥98% of the cells), whereas few expressed CD34 and CD45 (expressed by ≤4.12% of the cells) in all conditions (*n* = 4 biological replicates). Graphs show individual values and the mean ± SD.

**Figure 4:**
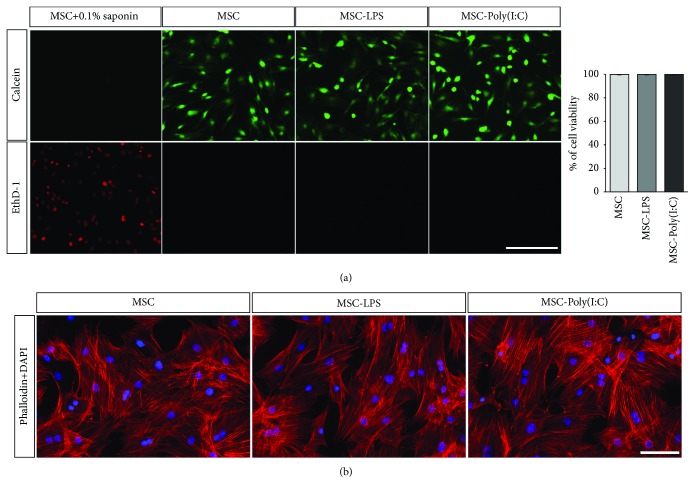
Assessment of the viability and morphology of unstimulated and preconditioned rBM-MSCs. (a) Representative photomicrographs showing the incorporation of calcein AM and its conversion into calcein (green) in live cells. Ethidium homodimer-1 (EthD-1; red) was used to label dying cells. Cells treated with 0.1% saponin were used as a positive control. Scale bar: 200 *μ*m. On the right, quantification of the percentage of live cells in each condition. Preconditioning with polyinosinic-polycytidylic acid (MSC-Poly(I:C)) or lipopolysaccharide (MSC-LPS) did not change cell viability in comparison to unstimulated rat bone marrow-derived mesenchymal stromal cells (MSC; *n* = 5 biological replicates). Bars represent the means ± SD. (b) Representative photomicrographs showing the morphology of phalloidin-stained rat bone marrow mesenchymal stromal cells (red) from the different experimental groups. Nuclear staining with 4′,6-diamidino-2-phenylindole (DAPI) in blue. Scale bar: 100 *μ*m.

Flow cytometry analysis was performed to determine whether the preconditioning protocols with LPS or Poly(I:C) would change the expression of key cell surface markers used for the identification of the MSC phenotype. Cells were treated with LPS or Poly(I:C) or kept in control medium without stimulation for 1 h, washed, and allowed to grow for an additional 23 h. While CD90 and CD29, two MSC markers [38, 39], were expressed by more than 98% of the cells in all conditions (Figure 3), less than 4.12% of the cells expressed the hematopoietic lineage markers CD45 and CD34 in all experimental groups (Figure 3). In line with these results, we observed that the morphology of MSCs was not changed 23 h after the removal of LPS or Poly(I:C), as assessed by staining the cells with phalloidin for F-actin labeling (Figure 4(b)). Moreover, the viability of rBM-MSCs was not affected by LPS or Poly(I:C), as demonstrated by the LIVE/DEAD viability assay performed 23 h after removal of the stimulus (Figure 4(a)). These results indicate that the preconditioning strategies employed in this study were not cytotoxic and did not induce changes in the expression profile of cell surface markers and in the morphology of rBM-MSCs.

### 3.3. Effects of LPS and Poly(I:C) Preconditioning on the Release of Inflammatory Mediators and VEGF by rBM-MSCs

**Figure 5:**
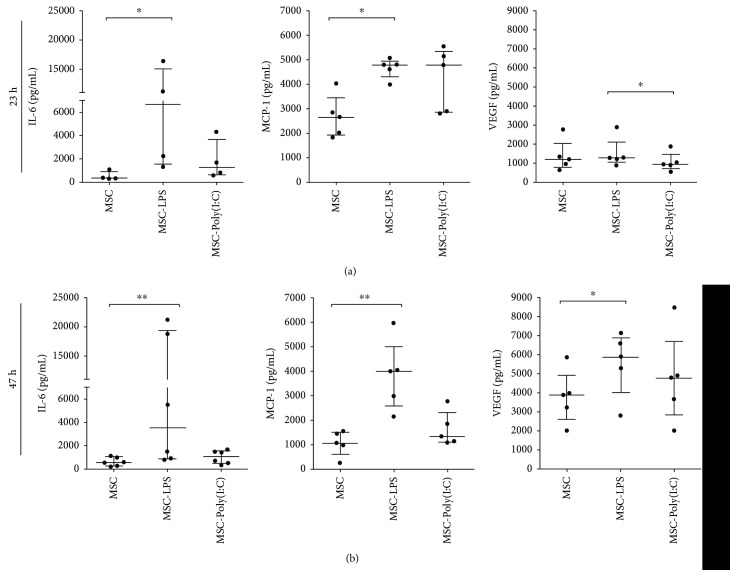
Evaluation of the secretion of bioactive factors by unstimulated and preconditioned rBM-MSCs. (a, b) Luminex® immunoassays were used to quantify the levels of interleukin- (IL-) 6, monocyte chemoattractant protein 1 (MCP-1), and vascular endothelial growth factor (VEGF) in the culture medium of rat bone marrow-derived mesenchymal stromal cells, 23 h (a) and 47 h (b) after a brief stimulation (1 h) with polyinosinic-polycytidylic acid (MSC-Poly(I:C)) or lipopolysaccharide (MSC-LPS), in comparison to unstimulated cells (MSC). Graphs show individual values and median with an interquartile range. ^∗^*p* < 0.05; ^∗∗^*p* < 0.01; the Friedman test with Dunn's multiple comparison test (*n* = 4–6 biological replicates, with each sample analyzed in duplicate).

MSCs have been shown to exert their therapeutic actions through paracrine mechanisms, including the release of trophic factors, cytokines, and chemokines [1, 40]. We therefore assessed whether a brief exposure to LPS or Poly(I:C) for 1 h would change the secretory capacity of rBM-MSCs. Using a magnetic luminex immunoassay, we measured the expression of soluble molecules in the conditioned medium of rBM-MSCs cultured for an additional 23 h or 47 h after the stimulus was removed. This analysis revealed that rBM-MSCs secreted IL-6 under standard culture conditions and that LPS stimulation increased the concentration of IL-6 in the conditioned medium at both time points, in comparison to unstimulated rBM-MSCs (Figure 5). Poly(I:C) had a stimulatory effect on the release of IL-6, which did not reach a statistical significance (Figure 5). Also, rBM-MSCs constitutively secreted VEGF, although it was not possible to quantitatively determine VEGF levels due to the high concentration of this molecule in all samples. Conversely, the levels of IFN-*γ*, IL-1*β*, IL-2, IL-4, IL-10, IL-13, IL-18, and TNF-*α* remained below the detection limit of the assay in all groups.

Considering that VEGF is involved in several reparative and regenerative processes, including angiogenesis, neurogenesis, and peripheral nerve regeneration [41, 42], we conducted another set of experiments to quantify VEGF levels in diluted samples (1 : 5) from preconditioned and unstimulated rBM-MSCs. VEGF levels were significantly higher in the conditioned medium from LPS-preconditioned rBM-MSCs, in comparison to the conditioned medium from unstimulated rBM-MSCs, at 47 h. In contrast, Poly(I:C) preconditioning did not increase VEGF secretion by rBM-MSCs. VEGF levels were even lower in the culture medium from Poly(I:C)-preconditioned rBM-MSCs than in the culture medium from LPS-preconditioned rBM-MSCs at 23 h (Figure 5). MCP-1 levels were also evaluated in this experiment, given the central role of this chemokine in Wallerian degeneration and axonal regeneration [43, 44]. We observed that MCP-1 was released by unstimulated rBM-MSCs and that LPS stimulation increased the concentration of this molecule in the culture medium at 23 h and 47 h. We also found higher levels of MCP-1 in the culture medium of Poly(I:C)-preconditioned rBM-MSCs at 23 h than in the culture medium from unstimulated rBM-MSCs, although without a statistical significance (Figure 5). Since the maintenance culture medium was supplemented with 10% FBS, we also analyzed the levels of VEGF and MCP-1 in fresh cell-free maintenance medium, to exclude the possibility that we were detecting VEGF and MCP-1 from the FBS. Both VEGF and MCP-1 levels were below the detection limit of the assay, which was validated for the detection of rat proteins. Taken together, these results showed that the physiological release of IL-6, MCP-1, and VEGF by cultured rBM-MSCs can be enhanced by a brief exposure to LPS, whereas the production of other proinflammatory (IFN-*γ*, IL-1*β*, IL-2, IL-18, and TNF-*α*) and anti-inflammatory cytokines (IL-4, IL-10, IL-13) was not observed in any conditions.

### 3.4. LPS Preconditioning Does Not Affect the Capacity of rBM-MSC-Conditioned Medium to Induce Neurite Growth in DRG Explants

**Figure 6:**
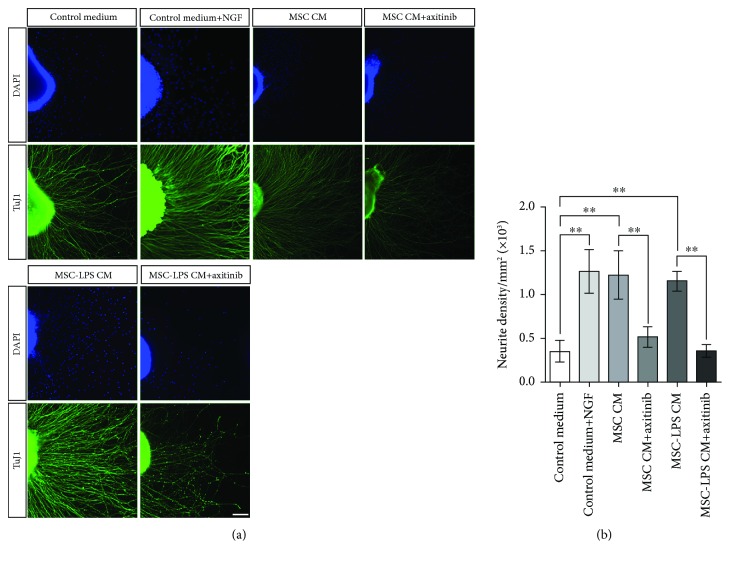
Analysis of the effect of the conditioned medium from unstimulated and LPS-preconditioned rBM-MSCs on neurite outgrowth. (a) Representative photomicrographs of dorsal root ganglion (DRG) explants immunolabeled with TuJ1 antibody (green) 24 h after incubation with fresh control medium supplemented with 5% fetal bovine serum (Control Medium group), bone marrow-derived mesenchymal stromal cell- (rBM-MSC-) conditioned medium (MSC CM group), rBM-MSC-conditioned medium+axitinib (a VEGF receptor inhibitor) (MSC CM+axitinib group), lipopolysaccharide- (LPS-) preconditioned rBM-MSC-conditioned medium (MSC-LPS CM group), or LPS-preconditioned rBM-MSC-conditioned medium+axitinib (MSC-LPS CM+axitinib group). Fresh control medium supplemented with 5% fetal bovine serum and nerve growth factor was used as a positive control (control medium+NGF group). Nuclear staining with 4′,6-diamidino-2-phenylindole (DAPI) in blue. Scale bar: 100 *μ*m. (b) Quantitative analysis of neurite density in DRG explants showing that LPS preconditioning did not affect the capacity of rBM-MSC-conditioned medium to induce neurite growth in dorsal root ganglion sensory neurons (*n* = 6 explants per group; rBM-MSCs from only 1 animal were used in this experiment to reduce donor-related variability). ^∗∗^*p* < 0.001; one-way ANOVA followed by the Newman-Keuls post hoc test.

We have previously shown that the paracrine activity of rBM-MSCs enhances neurite outgrowth in DRG sensory neurons in a transwell co-culture system [33]. Considering that rBM-MSCs constitutively secreted high levels of VEGF, as shown above, and that VEGF has been shown to promote neurite outgrowth in DRG sensory neurons [45, 46], we hypothesized that this neurotrophic activity of rBM-MSCs could be at least partially mediated by VEGF. To test this hypothesis, DRG explants were grown in DMEM/F12 control medium, DMEM/F12+rBM-MSC-conditioned medium, or DMEM/F12+rBM-MSC-conditioned medium+axitinib (a VEGF receptor inhibitor). DMEM/F12+NGF was used as a positive control. We observed that rBM-MSC-conditioned medium increased neurite density similarly to NGF and that axitinib abolished this effect (Figure 6). Given that LPS preconditioning increased the levels of VEGF in rBM-MSC culture medium, we also evaluated whether this preconditioning strategy would affect the neurotrophic activity of rBM-MSCs. Our results showed that LPS preconditioning did not change the effect of rBM-MSC-conditioned medium on the promotion of neurite growth in vitro and that axitinib abolished this effect (Figure 6).

### 3.5. Effect of LPS Preconditioning on the Proregenerative Ability of rBM-MSCs in the Peripheral Nervous System

**Figure 7:**
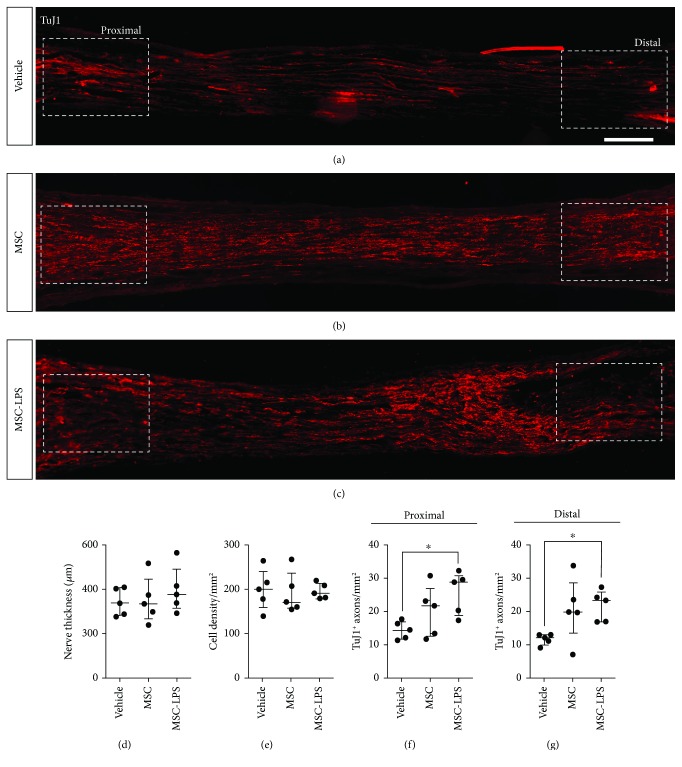
Analysis of the effect of LPS preconditioning on the proregenerative ability of rBM-MSCs. (a–c) Photomontages showing the regenerated portions of the sciatic nerve that were inside the polyethylene tubes 4 weeks after transection. Axons were stained with TuJ1 antibodies (red). In the Vehicle group, only the Matrigel® solution was injected into the tube, while in the MSC and LPS-MSC groups, 5 × 10^5^ rat bone marrow-derived mesenchymal stromal cells (rBM-MSCs) or lipopolysaccharide- (LPS-) preconditioned rBM-MSCs, respectively, were suspended in the Matrigel® solution and then injected into the tube immediately after sciatic nerve transection and tubulization. Scale bar: 200 *μ*m. (d–g) Quantitative analysis of nerve thickness, cell density, and number of TuJ1-positive axons (in the proximal and distal nerve segments) in each condition (*n* = 5 rats per group; rBM-MSCs from only 1 animal were used in this experiment to reduce donor-related variability). Data are shown as individual values, median, and interquartile range. ^∗^*p* < 0.05; Kruskal-Wallis test with Dunn's multiple comparisons test.

Considering that VEGF, IL-6, and MCP-1 are involved in peripheral nerve repair and regeneration [42, 43, 47], we evaluated the proregenerative capacity of rBM-MSCs and LPS-preconditioned rBM-MSCs in a rat model of sciatic nerve transection. Animals were divided into 3 groups. In the Vehicle group, only the Matrigel® solution was injected into the tube, while in the other two experimental groups, 5 × 10^5^ rBM-MSCs or LPS-preconditioned rBM-MSCs, respectively, were suspended in the Matrigel® solution and injected into the tube immediately after sciatic nerve transection and tubulization. Compared to the Vehicle group, treatment with LPS-preconditioned rBM-MSCs or unstimulated rBM-MSCs tended to increase the number of TuJ1^+^ axons in the proximal and distal portions of the regenerated nerve segment 4 weeks after the injury. In fact, there were statistically significant differences in the number of TuJ1^+^ axons per square millimeters, comparing animals that received the vehicle and those treated with LPS-preconditioned rBM-MSCs, in both the proximal and distal portions of the regenerated nerve segment (Figures 7(a)–7(c), 7(f), and 7(g)). No differences in nerve thickness or in cell density were observed among the groups (Figures 7(d) and 7(e)).

## 4. Discussion

BM-MSCs play a crucial role in the maintenance and regulation of hematopoietic stem cells and their progeny in the hematopoietic niche, and these regulatory actions can be influenced by PAMPs [48–51]. The therapeutic potential of MSCs from different sources may also rely on their capacity for activation by locally or systemically released factors that can modulate their secretory activities [52–54]. This evidence has led to the search of preconditioning strategies aimed at directing the paracrine activity of MSCs. Waterman and colleagues [20], for instance, have shown that a brief stimulation of human MSCs with low doses of LPS or Poly(I:C), two TLR agonists, can induce the so-called MSC1 and MSC2 phenotypes, respectively, analogously to the M1/M2 polarization of macrophages and the Th1/Th2 paradigm.

In the first part of the present study, we tested whether and how rBM-MSCs respond to Poly(I:C), using the protocol described by Waterman et al. [20]. We showed that rBM-MSCs express TLR3 and MDA5, two receptors for Poly(I:C), at the mRNA level. Then, we used confocal video microscopy to show, for the first time, the internalization of Poly(I:C) by MSCs. We demonstrated that the internalization of Poly(I:C) is a relatively rapid event, already observed within the first 15 min after the beginning of incubation. These results validated the use of this protocol (brief stimulation of rBM-MSCs with Poly(I:C) for 1 h) and are in agreement with previous studies reporting the expression of TLR3 mRNA by mouse and human BM-MSCs (mBM-MSCs and hBM-MSCs, respectively) [15, 55–58]. These findings are also in line with the work by Raicevic et al., who reported that hBM-MSCs express functional MDA5 [59]. In their study, Poly(I:C) internalization was facilitated by the transfection reagent LyoVec, an approach that has been shown to stimulate retinoic acid-inducible gene- (RIG-) I-like receptors (RLR), such as MDA5, rather than TLR3 [60, 61]. Although the activation of RLR can trigger cell death, Raicevic et al. showed that only prolonged stimulation (48 h) with high doses of Poly(I:C) in conjunction with LyoVec (but not with Poly(I:C) alone) can induce cell death in hBM-MSCs [59]. Our results agree with these findings, indicating that a brief exposure to Poly(I:C) for 1 h does not affect the viability of rBM-MSCs. Moreover, cell morphology and the expression of cell surface markers were not changed by this preconditioning protocol, suggesting that the cells kept their MSC identity after stimulation. Previous studies, however, have shown that Poly(I:C) stimulation increased the osteogenic and adipogenic differentiation potential of rBM-MSCs and hBM-MSCs in the presence of the appropriate inductive stimuli [27, 58, 62]. The expression of functional MDA-5 and TLR3 by BM-MSCs may therefore allow these cells to respond to double-stranded RNA from viruses without changing their MSC identity.

One of the main features of the MSC2 profile reported by Waterman et al. was the increased secretion of the chemokines IP-10 and RANTES and to a lesser extent the anti-inflammatory cytokines IL-4 and IL-10 [20]. We were not capable of reproducing these findings using rBM-MSCs, which did not secrete detectable levels of IL-4 or IL-10 in any conditions. IP-10 and RANTES, however, were not included in our screening assays. We nonetheless found that a brief stimulation with Poly(I:C) tended to increase the secretion of IL-6 and MCP-1 by rBM-MSCs, although these effects did not reach a statistical significance. In this regard, IL-6 was the cytokine whose induction was most consistently reported in previous preconditioning studies that have stimulated mBM-MSCs and hBM-MSCs with different doses and exposure times of Poly(I:C) (Table 2). This probably indicates that the dose employed in our study was too low or that the duration of stimulation was too brief. Most of the studies outlined in Table 2 have exposed MSCs to Poly(I:C) for at least 4 h, whereas we stimulated the cells with Poly(I:C) for only 1 h. It has also been demonstrated that human nasal mucosa-derived MSCs only secreted high levels of IL-6 and IL-8 following a prolonged (24 h) stimulation with Poly(I:C), revealing a mechanism of autocrine priming where the first cytokines that were released increased the sensitivity of MSCs to Poly(I:C) [63]. Accordingly, the dose employed in our study (1 *μ*g/mL) has been shown to induce the release of IL-6 by hBM-MSCs and mBM-MSCs in studies where the cells were stimulated for longer periods of time (Table 2).

The second protocol for rBM-MSC preconditioning tested in this study was a brief stimulation with LPS, a bacteria-derived molecule commonly used to induce a proinflammatory profile in different cell types [64]. We showed that rBM-MSCs expressed TLR4, the receptor for LPS, at the RNA level, which is in agreement with Shi et al. [65], as well as with studies performed with mBM-MSCs and hBM-MSCs [15, 55–58]. Then, we showed that a brief stimulation with LPS (using the protocol described by Waterman and colleagues [20]) did not change the viability of rBM-MSCs. In fact, previous studies have reported that LPS preconditioning protects mBM-MSCs from H_2_O_2_/serum deprivation-induced apoptosis in a TLR4- and MyD88-dependent manner [66] and prevents apoptosis in rBM-MSCs in response to hypoxia and serum deprivation [67]. Our results indicated that rBM-MSCs maintain their morphology and the expression of MSC cell surface markers after a brief stimulation with LPS. Interestingly, LPS preconditioning, in combination with TNF-*α* [68] or with the use of electrospun nanofibers [69], can enhance the osteogenic potential of BM-MSCs grown in osteogenic medium, although our data suggest that LPS does not change their MSC identity under normal culture conditions.

Waterman et al. have shown that human MSC1 cells are characterized by the capacity to secrete higher levels of IL-6 and IL-8 [20]. We therefore evaluated whether LPS changes the secretory function of rBM-MSCs. A literature search revealed that the increased secretion of IL-6 that we observed after LPS stimulation is a very consistent and reproducible response. The levels of IL-6 were reported to be elevated in many studies that had investigated the effects of LPS preconditioning in mouse and human BM-MSCs, regardless of the protocol used (Table 2). We could not find detectable levels of TNF-*α* or IL-1*β* in the supernatants of rBM-MSCs in any experimental conditions. This was a surprising finding, considering that Yan et al. and Ye et al. have shown that LPS induces the production of these two cytokines by rBM-MSCs [24, 25]. However, they used different protocols, including the use of a different rat strain, higher doses of LPS, and prolonged exposure times; also, Yan et al. [24] cultured the cells on titanium surfaces (Table 2). Moreover, the induction of IL-1*β* and TNF-*α* release was only reported by a few of the studies that have examined the effects of LPS on mBM-MSCs and hBM-MSCs (Table 2). In agreement with our findings, some of these studies have shown that MCP-1 and VEGF secretion is induced in mBM-MSCs and hBM-MSCs following stimulation with LPS (Table 2). The functional role of MSC-derived MCP-1 was reported by Shi and colleagues [49], who demonstrated that an intraperitoneal injection of low-dose LPS in mice increased the production of MCP-1 by BM-MSCs, which, in turn, was necessary for the mobilization of inflammatory monocytes into the bloodstream. It is possible that the induction of MPC-1 release by LPS preconditioning could be useful in situations where the production of this chemokine is impaired by the primary disease or a comorbidity of the patient who will be treated with autologous BM-MSCs, if this chemokine is necessary for the desired therapeutic effect. For instance, BM-MSCs from lupus-like mice and patients with systemic lupus erythematosus showed reduced levels of MCP-1, which impaired their capacity to suppress B-cell responses [70], and MCP-1 mRNA expression was decreased in BM-MSCs from patients with newly diagnosed type 1 diabetes [71]. Moreover, an interesting study found an inverse correlation between the release of IL-6, IL-8, and VEGF by BM-MSCs from multiple sclerosis patients and the number of white matter lesions in their brains [72].

We also showed that the release of VEGF was increased after LPS preconditioning, although in a delayed manner. A possible explanation for this delay could be an autocrine response of rBM-MSCs to mediators released in the first hours after stimulation. Other studies have also found that mBM-MSCs and hBM-MSCs release higher levels of VEGF in response to LPS (Table 2), proinflammatory cytokines, or growth factors [73–77]. A recent study has found that soluble oligomers of the amyloid-*β* peptide (A*β*Os) increased the levels of VEGF in the culture medium of rBM-MSCs and that these cells protected neurons from A*β*O-induced oxidative stress in a coculture system, an effect that was prevented by a cocktail of neutralizing antibodies against IL-6, IL-10, and VEGF [78].

Notably, all the soluble factors whose secretion was influenced by LPS in the present study were already constitutively secreted by rBM-MSCs, suggesting again that the cells retained their MSC identity. We also noted a considerable variability in the magnitude of the secretory response to LPS and Poly(I:C), which reflects the challenge of developing MSC-based immunomodulatory products with predicted efficacy [79]. The levels of secreted factors, however, could be used as biomarkers to predict the efficacy of MSCs, as demonstrated by Kim et al., who found a correlation among the levels of four factors (VEGF, MCP-1, IL-8, and angiogenin) and the proangiogenic activity of human Wharton's jelly-derived MSCs from different donors [80].

Then, we showed that the conditioned medium from rBM-MSCs increased neurite outgrowth in DRG explant cultures, in agreement with previous findings from experiments using the conditioned medium from hBM-MSCs [81] or rBM-MSCs in a coculture transwell system [33]. The very high levels of VEGF in the conditioned medium from unstimulated MSCs may explain why there was no difference between the effects of the conditioned medium from unstimulated and LPS-stimulated rBM-MSCs on the enhancement of neurite outgrowth. This hypothesis is supported by the absence of trophic activity following VEGF-receptor inhibition in both experimental conditions, suggesting that VEGF is a crucial factor for the effects of rBM-MSCs and LPS-preconditioned rBM-MSC-conditioned medium on DRG neurons. In addition, the VEGFR1 receptor is expressed by peripheral sensory neurons in cancer pain as well as by spinal cord motoneurons during development and regeneration [82]. Finally, we found that LPS-preconditioned MSCs increased the number of regenerating axons in a model of sciatic nerve transection. Although the mechanisms behind this proregenerative effect were not investigated in the present study, current evidence indicates that the paracrine activity of MSCs is largely responsible for most of their therapeutic activities in the peripheral nervous system [33, 83]. All three factors that were constitutively secreted by rBM-MSCs and whose secretion was enhanced by LPS (VEGF, IL-6, and MCP-1) are important mediators of inflammatory and regenerative processes that take place in injured peripheral nerves. Macrophage-derived VEGF, for example, is responsible for the formation of new blood vessels that are used as scaffolds for the migration of Schwann cells that guide regenerating axons [42]. In turn, IL-6 signaling accelerates nerve regeneration [47] and MCP-1 is necessary for the recruitment of macrophages to injured nerves during Wallerian degeneration [84, 85]. Implantation of an atelocollagen sponge containing MCP-1 and the secreted ectodomain of sialic acid-binding Ig-like lectin-9 promoted the polarization of macrophages into the tissue-repairing M2 phenotype and restored nerve function in a model of facial nerve transection in rats [86]. In addition, IL-6 secretion was shown to be necessary for the immunomodulatory effects of mBM-MSCs (primed with proinflammatory cytokines) on macrophage polarization in vitro [87]. We cannot neglect, however, the contribution of other mechanisms to the beneficial effects of rBM-MSCs on nerve regeneration. The immunosuppressive function of MSCs, for instance, has been explored for the treatment of several autoimmune disorders [88] and may counterbalance the deleterious effects of neuroinflammation. The release of extracellular vesicles by MSCs [89] and the transfer of healthy organelles from MSCs to dysfunctional or dying cells [90, 91] are also the focus of intensive research. A detailed functional and mechanistic characterization of the effects of preconditioned rBM-MSCs on nerve regeneration was beyond the scope of the present study but surely deserves further investigation.

## 5. Conclusions

rBM-MSCs are capable of internalizing Poly(I:C), but the magnitude of their paracrine response to a brief stimulation with Poly(I:C) varied greatly, a limitation that should be addressed in future studies. LPS preconditioning boosted the production of constitutively secreted factors by rBM-MSCs, in accordance with previous findings in human and mouse BM-MSCs. Importantly, these effects were achieved without changing the mesenchymal stromal cell identity of the cells or impairing their neurotrophic actions. Our results support the utilization of rBM-MSCs for the investigation of preconditioning strategies aimed at improving the efficacy of MSCs-based therapies, especially in preclinical studies in rats, which are still widely used in the field of neural regeneration [3, 28, 30, 83, 92, 93].

## Figures and Tables

**Table 1:** Primers used for RT-PCR.

Name	Primer	Sequence
TLR3	Forward	TCTGCACGAACCTGACAGAG
	Reverse	CAGTTGGACCCAAGTTCCCA
TLR4	Forward	TCTCACAACTTCAGTGGCTGG
	Reverse	AGTACCAAGGTTGAGAGCTGG
MDA5	Forward	TCCGGGAAGGTTATCGTCCT
	Reverse	GGGTATCGCCGCTTAATCCA
CypA	Forward	TATCTGCACTGCCAAGACTGAGTG
	Reverse	CTTCTTGCTGGTCTTGCCATTCC
GAPDH	Forward	CAACTCCCTCAAGATTGTCAGCAA
	Reverse	GGCATGGACTGTGGTCATGA

**Table 2:** Effects of LPS and Poly(I:C) on the secretory capacity of BM-MSCs; a literature review summary.

Cell type (source)	Agonist (dose and exposure time)	Secreted proteins induced by LPS	Secreted proteins induced by Poly(I:C)	References
rBM-MSCs (Sprague Dawley rats) cultured on titanium surface	LPS (1 *μ*g/mL for 6–72 h)	TNF-*α*, IL-1*β*	NA	[24]
rBM-MSCs (Sprague Dawley rats)	LPS (1 *μ*g/mL for 6 h)	TNF-*α*, IL-1*β*	NA	[25]
mBM-MSCs (C57BL/6J mice)	LPS or Poly(I:C) (20 *μ*g/mL for 24–72 h or 10 ng/mL–20 *μ*g/mL for 7 days)	IL-6	IL-6	[15]
mBM-MSCs (C57BL/6J mice)	LPS (100 ng/mL for 24 h)	IL-6, VEGF-A	NA	[73, 74]
mBM-MSCs (C57BL/6 mice)	LPS (1 *μ*g/mL for 48 h) or Poly(I:C) (20 *μ*g/mL for 48 h)	IL-6	IL-6	[56]
mBM-MSCs (C57BL/10J mice)	LPS (0.01 to 10 *μ*g/mL for 48 h)	VEGF	NA	[22]
mBM-MSCs (C57BL/6J mice)	LPS (1 *μ*g/mL for 24 h) or Poly(I:C) (50 *μ*g/mL for 24 h)	IL-6, IL-1*β*, IFN-*γ*	IL-6, TGF-*β*, IL-10, IL-1*β*, and IFN-*β*	[58]
mBM-MSCs (C57BL/6J mice)	LPS (0.01–10 mg/mL for 24 h) or Poly(I:C) (0.01–10 mg/mL for 24 h)	IL-6	IL-6	[94]
mBM-MSCs (C57BL/6J mice)	LPS (1000 ng/mL for 3 days)	IL-6, IL-1*β*	NA	[95]
hMSCs	LPS (10 ng/mL for 24 or 48 h) or Poly(I:C) (1 *μ*g/mL for 24 or 48 h)	IL-6, IL-8, IP-10, TNF-*α*	IL-10, IL-12	[19]
hMSCs	LPS (10 ng/mL for 1 h) or Poly(I:C) (1 *μ*g/mL for 1 h)+48 h without stimuli	IL-6, IL-8	IP-10, RANTES	[20]
hBM-MSCs	LPS (10 *μ*g/mL for 18 h) or Poly(I:C) (30 *μ*g/mL for 18 h)	IL-6, iL-8, RANTES	IL-6, IL-8, RANTES	[96]
hBM-MSCs	LPS (10 *μ*g/mL for 48 h)	IL-6, MCP-1, IL-8, TNF-*α*, GM-CSF, IL-1*β*, G-CSF, MIP-1*β*, IFN-*γ*, IL-17, and IL-4	NA	[97]
hBM-MSCs	Poly(I:C) (1 *μ*M for 4–24 h)	NA	IL-6, IL-8, IFN type I	[63]
hBM-MSCs	LPS (1–1000 ng/mL for 48 h) or Poly(I:C) (0.02–20 *μ*g/mL for 48 h)	IL-6, IL-8, RANTES, IL-1*β*	IL-6, IL-8, RANTES, IL-1*β*	[56]
hBM-MSCs	LPS (5 *μ*g/mL for 24 h) or Poly(I:C) (50 *μ*g/mL for 24 h)	IL-6	IL-6	[57]
hBM-MSCs	LPS (10 *μ*g/mL for 18 h) or Poly(I:C) (30 *μ*g/mL for 18 h)	IL-6	IL-6, IL-23	[16]
hBM-MSCs	LPS (100 ng/mL for 24 h) or Poly(I:C) (100 *μ*g/mL for 24 h)	IL-6, IL-8, GM-CSF	IL-6, IL-8, GM-CSF, and IFN-*β*	[98]
hBM-MSCs	LPS (10 ng/mL for 4 h) or Poly(I:C) (1–5 *μ*M/mL for 4 h)+24 h without stimuli	IL-6, IL-8, IP-10, RANTES, and IFN-*α*	IL-6, IL-8, IP-10, RANTES, and IFN-*α*	[99]
hBM-MSCs	Poly(I:C) (5 *μ*g/mL for 24 h)	NA	IL-6, IP-10	[100]
hBM-MSCs	LPS (10 *μ*g/mL for 48 h)	IL-6, GM-CSF, G-CSF, M-CSF, and IL-11	NA	[101]
hBM-MSCs	LPS (10 ng/mL for 24 h)	IL-6, VEGF, MCP-1, IL-8, IP-10, RANTES, GM-CSF, MIP-1*α*, MIP-1*β*, SDF-1*α*, IL-1RA, GRO-*α*, IL-1*α*, IL-2, IL-31, and eotaxin	NA	[77]

G-CSF: granulocyte colony-stimulating factor; GM-CSF: granulocyte-macrophage colony-stimulating factor; GRO-*α*: growth-regulated oncogene-*α*; hBM-MSCs: human bone marrow-derived mesenchymal stromal cells; hMSCs: human mesenchymal stromal cells (unclear origin); IFN: interferon; IL: interleukin; IP-10: interferon gamma-induced protein 10; LPS: lipopolysaccharide; mBM-MSCs: mouse bone marrow-derived mesenchymal stromal cells; MCP-1: monocyte chemoattractant protein-1; M-CSF: macrophage colony-stimulating factor; MIP: macrophage inflammatory protein; NA: not available; Poly(I:C): polyinosinic-polycytidylic acid; RANTES: regulated on activation, normal T cell expressed and secreted; rBM-MSCs: rat bone marrow-derived mesenchymal stromal cells; VEGF: vascular endothelial growth factor.

## Data Availability

The data used to support the findings of this study are available from the corresponding author upon request.
