# Erratum to “Preconditioning of Rat Bone Marrow-Derived Mesenchymal Stromal Cells with Toll-Like Receptor Agonists”

**DOI:** 10.1155/2020/5857046

**Published:** 2020-08-21

**Authors:** Fabiana Evaristo-Mendonça, Gabriela Sardella-Silva, Tais Hanae Kasai-Brunswick, Raquel Maria Pereira Campos, Pablo Domizi, Marcelo Felippe Santiago, Ricardo Augusto de Melo Reis, Rosalia Mendez-Otero, Victor Túlio Ribeiro-Resende, Pedro Moreno Pimentel-Coelho

**Affiliations:** ^1^Instituto de Biofísica Carlos Chagas Filho, Universidade Federal do Rio de Janeiro, Rio de Janeiro, RJ 21941-902, Brazil; ^2^Centro Nacional de Biologia Estrutural e Bioimagem (CENABIO), Universidade Federal do Rio de Janeiro, Rio de Janeiro, RJ 21941-902, Brazil; ^3^Instituto Nacional de Ciência e Tecnologia em Medicina Regenerativa, Rio de Janeiro, RJ 21941-902, Brazil; ^4^Núcleo Multidisciplinar de Pesquisa em Biologia (Numpex-Bio), Campus de Duque de Caxias Geraldo Guerra Cidade, Universidade Federal do Rio de Janeiro, Duque de Caxias, RJ 25255-030, Brazil

In the article titled “Preconditioning of Rat Bone Marrow-Derived Mesenchymal Stromal Cells with Toll-Like Receptor Agonists” [1], there was an error in the production of Figure 2(b) which resulted in some colouration being lost. The publisher apologises for introducing this error, and the corrected figure is shown below and is listed as Figure 1:

## Figures and Tables

**Figure 1:**
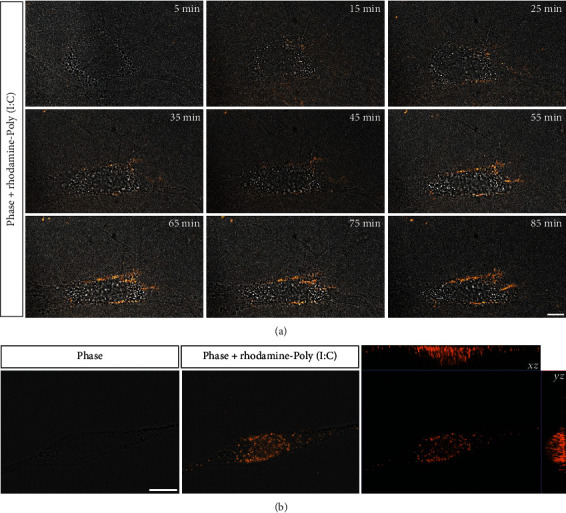
Internalization of rhodamine-conjugated polyinosinic-polycytidylic acid (Poly(I:C)) by rBM-MSCs. (a) Representative photomicrographs from time-lapse video microscopy, each corresponding to the indicated time points following the addition of rhodamine-conjugated Poly(I:C) to the culture medium, showing the overlay of phase contrast and spinning disk confocal fluorescence images of one cell (rhodamine-conjugated Poly(I:C) is shown in red). Scale bar: 10 *μ*m. (b) On the left, phase contrast image of a cell at 1 h after incubation with rhodamine-conjugated Poly(I:C). On the center, overlay of the phase contrast image and the maximum intensity projection of a z stack showing rhodamine-conjugated Poly(I:C) in red. On the right, orthogonal projections (XZ, YZ) of the z stack showing the internalization of rhodamine-conjugated Poly(I:C) (in red) by this cell. Scale bar: 10 *μ*m.
